# A single-blind randomized controlled trial of ultrasound-guided Canggui Tanxue needling technique for contractural facial synkinesis

**DOI:** 10.1097/MD.0000000000049719

**Published:** 2026-07-17

**Authors:** Yuan Zhou, Yanru Wang, Xiaoming Wu, Xi Zhou, Linjia Wang, Jingxin Mao

**Affiliations:** a Departments of Acupuncture and Ultrasound, Chongqing Traditional Chinese Medicine Hospital, Chongqing, China; b Department of Rehabilitation, The Ninth People’s Hospital of Chongqing, Chongqing, China; c Technology Industry Development Center, Chongqing Medical and Pharmaceutical College, Chongqing, China

**Keywords:** acupuncture treatment, Canggui Tanxue acupuncture, contractural facial synkinesis, high-frequency ultrasound, shear wave elastography, Young modulus

## Abstract

**Background:**

Facial spastic synkinesis (FSS) is a disabling sequela of facial nerve palsy, with current treatments limited by transient efficacy, severe side effects, poor precision, and lack of quantifiable targets/objective evaluation. This study used ultrasound shear wave elastography combined with Canggui Tanxue needling to precisely locate lesioned muscles, exploring a safer, more effective targeted strategy to address the gap of insufficient precision and quantitative evidence in clinical practice.

**Methods:**

A single-blind randomized controlled trial was conducted at Chongqing Traditional Chinese Medicine Hospital’s Acupuncture Department (June 2022 to May 2023), enrolling 64 FSS patients randomized into an ultrasound-guided group (n = 31) and a control group (n = 33). The former received Canggui Tanxue needling at hypertonic muscles (Young modulus > 10 kPa) under real-time shear wave elastography, the latter received traditional syndrome differentiation-based acupuncture. Both groups had 6 sessions (twice weekly for 3 weeks). Outcome measures included primary (Young modulus of target muscles) and secondary (House–Brackmann grading, Facial Disability Index, and total effective rate) indicators. SPSS 26.0 was used for statistical analysis.

**Results:**

A total of 64 patients aged 27 to 66 years were enrolled, including 25 males (39.06%) and 39 females (60.94%). Baseline characteristics were comparable between groups (*P* > .05). Post-intervention, the ultrasound-guided group had significantly larger reductions in Young modulus of depressor anguli oris (3.00 vs 1.10 kPa) and levator labii superioris (5.60 vs 1.00 kPa) versus controls (*P* < .001). It also showed superior facial nerve recovery (House–Brackmann grading: *P* = .013), a higher total effective rate (87.1% vs 42.4%, *P* < .001), and greater improvement in physical function scores (74.84 ± 8.42 vs 68.94 ± 13.68, *P* = .04). Psychosocial function scores decreased in both groups (*P* = .92). Only 2 mild subcutaneous hematomas occurred in the ultrasound-guided group, confirming good safety.

**Conclusions:**

Ultrasound-guided Canggui Tanxue needling precisely targets hypertonic muscles, reduces pathological tension, improves FSS patients’ facial function, and is safe, offering an effective targeted strategy. Clinical promotion of this precise acupuncture technology is recommended, especially for late-stage facial nerve palsy patients with abnormal muscle tension. Future studies should extend follow-up to assess long-term efficacy/recurrence and combine neurophysiological/multimodal imaging to clarify its mechanism.

## 1. Introduction

Contractural facial synkinesis is a debilitating sequela of facial paralysis characterized by involuntary, abnormal co-contraction of facial muscles during voluntary movements, often manifesting as paradoxical deviation of the oral commissure towards the affected side during the later stages of facial paralysis.^[1,2]^ Facial paralysis is a common neurological disorder with a global distribution, and its incidence varies across different regions. Such regional differences are closely associated with factors like ethnic background and environmental conditions.^[3]^ Among patients with facial paralysis, parts of them will develop facial spastic synkinesis (FSS), and the incidence can reach 55% particularly in those whose condition remains unresolved for more than 3 months.^[4]^ This condition typically arises from prolonged unresolved facial muscle paralysis, where muscles transition from a flaccid state to a stiff and spastic condition. It can lead to functional impairments in eating, drinking, and facial expression control, along with disfiguring appearance, significantly compromising patients’ psychological well-being and quality of life.^[5,6]^ Its pathological mechanisms remain incompletely elucidated but may involve aberrant reinnervation, where regenerating nerve fibers erroneously enter adjacent pathways to misinnervate effectors, or fibrosis of affected facial muscles and abnormal increases in muscle tension following neural regeneration.^[7]^

Current mainstay treatments for facial synkinesis, such as botulinum toxin injections, offer symptomatic relief but are transient, require repeated administrations, and do not address the underlying biomechanical abnormalities. Pharmacological interventions (e.g., corticosteroids), physical therapies (e.g., laser, infrared radiation), and surgical approaches (e.g., facial nerve decompression, botulinum toxin injections) may partially alleviate neural inflammation and edema.^[8–12]^ However, they are generally associated with transient effectiveness, significant side effects, or surgical risks, resulting in suboptimal patient compliance.^[13,14]^ While microvascular decompression represents a potentially curative option, it frequently entails postoperative inefficacy, recurrence, and complications such as hearing impairment.^[15]^ Consequently, exploring safer and more effective therapeutic strategies is imperative.

Acupuncture demonstrates potential in managing sequelae of facial paralysis. Substantial clinical evidence confirms that acupuncture can facilitate facial nerve functional recovery and ameliorate symptoms such as facial muscle spasms and synkinesis.^[6]^ Diverse techniques, including conventional acupuncture (e.g., Chifeng Yingyuan acupuncture), electroacupuncture, fire acupuncture, catgut embedding, and emerging technologies (e.g., laser acupoint irradiation), have been applied clinically with modest success. Li et al found that compared with transcutaneous electrical stimulation therapy, dense-sparse wave electroacupuncture could significantly improve facial symmetry in patients with chronic Bell palsy, and this therapeutic advantage was maintained throughout the follow-up period.^[16]^ Similarly, Zhou et al explored the efficacy of ultrasound-guided acupoint stimulation for synkinesis management, noting enhanced facial nerve conduction velocity and improved patient-reported outcomes compared to conventional acupuncture.^[17]^ However, existing evidence indicates that the therapeutic effects of such acupuncture-based interventions are often transient: a systematic review by Guntinas-Lichius et al revealed that 35 to 45% of patients experience symptom recurrence within 3 to 6 months posttreatment, with recurrence rates escalating to over 60% in those followed for more than 12 months.^[18]^ This highlights the unmet need for more durable, targeted therapeutic strategies in the management of facial synkinesis. Moreover, prolonged intensive stimulation of affected facial areas fails to specifically address synkinesis, presenting limitations and risks. As facial paralysis with “paradoxical deviation” involves structural alterations and localized tension changes in muscles, high-intensity acupuncture or electroacupuncture on the affected side may cause tissue reinjury, provoking nerve irritation and facial spasms. Alternatively, it could damage regenerating nerve fibers during repair, leading to aberrant regeneration syndromes (e.g., crocodile tears) or adhesion formation during tissue healing, resulting in synkinesis and even facial muscle atrophy/hypertrophy.^[19,20]^ Furthermore, evaluation systems lack objectivity, relying primarily on subjective scales like House–Brackmann (H–B) grading and Facial Disability Index (FDI) questionnaires rather than objective quantitative indicators reflecting local muscle contracture severity and biomechanical property changes (e.g., stiffness).^[21]^ This impedes therapy optimization and mechanistic exploration, particularly limiting the potential of techniques like Canggui Tanxue acupuncture, which requires precise stimulation of specific deep muscle layers.^[22]^

## 2. Materials and methods

### 2.1. Study design

#### 2.1.1. Study type

This was a single-blind randomized controlled trial (RCT) with a 1:1 random allocation ratio. A total of 64 patients were enrolled in the present study, including 25 males (39.06%) and 39 females (60.94%). The age of the participants ranged from 27 to 66 years old.

#### 2.1.2. Blinding implementation

According to the definition of blinding in clinical trials, this study adopted a single-blind design.

Blinding of participants: All participants were unaware of their group assignments. Before treatment, they were informed that 2 different acupuncture strategies would be used in the study, but no specific information about the intervention methods was disclosed. The treatment environment, duration, and basic operation procedures were kept consistent between the 2 groups to avoid unblinding.

Blinding of outcome assessors: Assessors responsible for shear wave elastography (SWE) testing, H–B grading, and FDI scale assessment did not participate in any treatment procedures. They only received coded data during assessment and were prohibited from communicating with treating acupuncturists about participant conditions.

Blinding of statisticians: Statistical analysts were provided with de-identified datasets for data analysis. Group decoding was performed only after the completion of all statistical tests to ensure the integrity of blinding.

All participant data were identified only by unique codes throughout the study, and no group-specific marks were made in medical records, SWE reports, or scale forms to prevent unblinding. This single-blind design is consistent with the blinding definition which reported by Day and Altman.^[23]^

#### 2.1.3. Randomization method

A randomization sequence was generated using SAS 9.4 software. Allocation concealment was implemented via sequentially numbered, opaque sealed envelopes. Envelope opening and group assignment were performed by researchers independent of the treatment procedures to minimize selection bias.

#### 2.1.4. Trial period

From June 2022 to May 2023, encompassing 3 phases: participant recruitment, intervention implementation, and outcome assessment. The intervention duration was 3 weeks (a total of 6 treatment sessions), with outcome measures collected immediately before treatment initiation and immediately after the completion of all treatment sessions.

### 2.2. Characteristics of the research setting

#### 2.2.1. Institution name

Department of Acupuncture and Moxibustion, Chongqing Hospital Traditional Chinese Medicine.

#### 2.2.2. Ethical statement

The present study was approved by the Medical Ethics Committee of Chongqing Traditional Chinese Medicine Hospital (approval No. 2022-ky-36). The study was retrospectively registered with the International Traditional Medicine Clinical Trial Registry (https://itmctr.ccebtcm.org.cn/mgt/project/view/6546064569585036874; ITMCTR2025000783). All procedures adhered to the Declaration of Helsinki. Before participating, all participants received detailed information about the study phases and required written consent for their participation.

#### 2.2.3. Suitability statement

As a key specialty of a grade a tertiary traditional Chinese medicine hospital, this department has extensive clinical experience in acupuncture treatment for facial nerve disorders, with an annual attendance of over 1000 patients with facial paralysis and its sequelae. It is equipped with a supersonic imagine aixplorer ultrasound diagnostic system, which can meet the requirements for real-time SWE-guided localization. Additionally, the department has independent acupuncture treatment rooms and standardized assessment areas, enabling strict control of the consistency of treatment procedures and outcome assessments. These conditions fully comply with the standardization and normalization requirements for research settings in RCT studies.

### 2.3. Participant recruitment process

#### 2.3.1. Recruitment channels

Participants were recruited through 4 approaches: hospital outpatient announcements, screening of the department’s medical record system, community health education, and follow-up of previous patients.

#### 2.3.2. Screening process

Preliminary screening, 2 trained physicians conducted outpatient interviews and physical examinations to initially determine whether participants met the diagnostic criteria for facial synkinesis and basic requirements such as age and disease duration. Standard reassessment: For those who initially met the criteria, further verification of the inclusion/exclusion criteria was performed (e.g., medical history collection and laboratory tests to rule out diabetes mellitus, coagulation disorders, etc), and baseline SWE testing was completed. Informed consent: eligible participants who met all criteria were fully informed of the study protocol, potential risks, and benefits. After signing a written informed consent form, they were enrolled in the randomization process.

#### 2.3.3. Recruitment period

Form June 2022 to March 2023, a total of 67 participants were screened over a 10-month period.

### 2.4. Study participants

#### 2.4.1. Inclusion criteria

Meeting the diagnostic criteria for facial neuritis issued by the neurology branch of the Chinese medical association in 2016, with clinically confirmed synkinesis; aged 16 to 75 years; time since onset of facial paralysis > 3 months; unilateral paralytic sequelae, with H–B grading ≥ grade II; and signed informed consent form.

#### 2.4.2. Exclusion criteria

Pseudoparalytic facial deviation; bilateral peripheral/central facial paralysis; facial paralysis secondary to surgery, trauma, intracranial lesions, or guillain-barré syndrome; severe systemic diseases (e.g., diabetes mellitus and Cushing syndrome), moderate-to-severe osteoporosis, uncontrolled hypertension or mental disorders; coagulation disorders or bleeding tendencies; and pregnancy/lactation period.

#### 2.4.3. Sample size explanation

A total of 67 patients were initially randomized. Among them, 1 patient was excluded due to failure to meet the inclusion criteria (secondary to postoperative intracranial tumor), and 2 patients withdrew due to refusal to participate in the study. Finally, 64 patients were included in the statistical analysis (31 in the ultrasound-guided group and 33 in the control group). All enrolled participants completed all 6 treatment sessions and outcome assessments without any dropouts during the study period.

### 2.5. Intervention measures

#### 2.5.1. Control group

Conventional acupuncture based on meridians/acupoints combined with the facial “three-line method” was adopted. Core meridians and acupoints included “Baihui” (GV20), “Fengfu” (GV16), “Taichong” (LR3), “Hegu” (LI4), “Shenting” (GV24), “Taiyang” (EX-HN5), “Xiaguan” (ST7), “Yifeng” (TE17), “Zusanli” (ST36), and “Neiting” (ST44), with a needling depth of 0.8 to 1 cun and the even reinforcing-reducing manipulation. The acupoint distribution of the facial “three-line method” was as follows: Midline acupoints (“Shenting” [GV24], “Yintang” [EX-HN3], “Shuigou” [GV26], and “Chengqiang” [CV24]); Paramedian line acupoints (approximately 1 cun lateral to the midline, including “Yangbai” [GB14], “Yuyao” [EX-HN4], “Chengqi” [ST1], “Sibai” [ST2], “Juliao” [ST3], and “Dicang” [ST4]); and Lateral line acupoints (“Taiyang” [EX-HN5], “Xiaguan” [ST7], and “Jiache” [ST6]). Auxiliary needling: For patients with nasal dysfunction, penetrating needling from “Yingxiang” (LI20) to “Neiyingxiang” (EX-HN8) was used; for those with mentalis muscle abnormalities, “Jiachengqiang” (EX-HN1) was selected. Facial acupoints were punctured with 0.5 to 1.5 cun needles. After deqi (needling sensation), an electroacupuncture apparatus was connected to deliver continuous wave stimulation, with needles retained for 30 minutes. Treatments were administered twice a week for 3 weeks (a total of 6 sessions) by the same experienced acupuncturist.

#### 2.5.2. Ultrasound-guided group

Canggui Tanxue needling was performed under real-time high-frequency ultrasound guidance. Localization procedure: before each treatment, a supersonic imagine aixplorer ultrasound diagnostic system (equipped with a linear S15-4 probe, frequency range of 1–16 MHz) was used for lesion localization. The patient was placed in a supine position with relaxed facial muscles. Conventional B-mode ultrasound scanning was conducted, followed by activation of the SWE mode. Regions with abnormally increased Young modulus (> 10 kPa), perimuscular hyperechogenicity, and blurred structure in the levator labii superioris, depressor anguli oris, and depressor labii inferioris muscles on the affected side were identified. A region of interest with a diameter of 7 to 10 mm was set on the target muscle, and the elastic range was preset to 0 to 460 kPa.

Needling procedure: The acupuncturist adopted the Canggui Tanxue needling technique. After fixing the marked target skin area with the left thumb, a disposable filiform needle was perpendicularly inserted into the lesion site according to the thickness of the facial muscles, with a needling depth of 0.5 to 1.0 cun. Deqi determination criteria: Deqi was defined as the occurrence of local soreness, distension, numbness, or heavy sensation in the participant, or a heavy, tight, or stagnant sensation under the needle felt by the acupuncturist. If the above manifestations did not appear after needling, the insertion depth or direction was adjusted until deqi was achieved. After deqi, the needle was withdrawn to the superficial layer (without exiting the skin), and then the needle direction was adjusted upward, downward, leftward, and rightward respectively. For each direction, a layered progressive insertion method (“superficial layer → middle layer → deep layer”) was adopted, simulating the movement of a “tortoise burrowing into the soil.” After multidirectional exploration, the needle was retained in the deep layer for 30 minutes. During needle withdrawal, the same operation was repeated at the original acupoint. Two complete sets of operational procedures were performed for each lesion target per treatment (1 set during needle insertion and 1 set during withdrawal). The treatment frequency, course, and operating acupuncturist were consistent with those in the control group.

### 2.6. Data collection

#### 2.6.1. Baseline data

Including demographic characteristics (gender and age), disease-related characteristics (affected side, disease duration, and baseline H–B grading), baseline Young modulus values of target muscles (measured SWE), and baseline FDI scores (FDI physical [FDIP] and FDI social [FDIS] subscales). Data were sourced from outpatient medical records, SWE examination reports, and scale completion records.

#### 2.6.2. Intervention process data

Treatment operation parameters (acupoints, needling depth, deqi status, and needle retention time), and adverse event data (type, onset time, management measures, and outcome). Data were sourced from treatment operation records and adverse event monitoring forms, which were recorded in real time by the treating acupuncturist.

#### 2.6.3. Outcome measure data

Primary outcome measure: Posttreatment Young modulus values of target muscles (levator labii superioris, depressor anguli oris, and depressor labii inferioris), with data sourced from SWE examination reports; Secondary outcome measures: Posttreatment H–B grading (physician assessment records), total clinical effective rate (calculated based on improvements in H–B grading), and posttreatment FDI scores (FDIP and FDIS subscales, completed by participants), with data sourced from outcome assessment records. All data were entered into an Excel database by a designated person and double-checked by 2 independent researchers to ensure accuracy.

### 2.7. Outcome measures

#### 2.7.1. Primary efficacy outcome measure

Young modulus values (unit: kPa) of the levator labii superioris, depressor anguli oris, and depressor labii inferioris muscles on the affected side. Measured via high-frequency ultrasound SWE, the absolute reduction value (pretreatment value minus posttreatment value) was calculated. A larger reduction value indicates a more significant improvement in muscle tension.

#### 2.7.2. Secondary efficacy outcome measures

Facial nerve function, assessed using the H–B grading system, which classifies function into grade I (normal) to grade VI (complete paralysis); total clinical effective rate: categorized based on improvements in H–B grading as follows: invalid (improvement < 1 grade), effective (improvement of 1 grade), markedly effective (improvement ≥ 2 grades), and cure (recovery to grade I). The total clinical effective rate was calculated using the formula: ([Number of effective cases + number of markedly effective cases + number of cured cases]/total number of cases) × 100%; quality of life: evaluated using the FDI, which includes the physical function subscale and social function subscale. A higher FDIP score indicates better physical function, while a lower FDIS score indicates a lighter psychosocial burden.

### 2.8. Safety outcome measures

Record the occurrence, severity, management, and outcome of adverse events (e.g., subcutaneous hematoma, nerve injury, vascular injury, etc) during the treatment process.

### 2.9. Statistical power analysis

Statistical analysis was performed using SPSS 26.0 software. For continuous variables, normality was first tested via the Shapiro–Wilk test: data conforming to a normal distribution were expressed as “mean ± standard deviation (mean ± SD),” with paired *t*-tests used for intragroup comparisons and independent samples *t*-tests for intergroup comparisons; data not conforming to a normal distribution were expressed as “median (interquartile range) [median (IQR)],” with Wilcoxon signed-rank tests used for intragroup comparisons and Mann–Whitney *U* tests for intergroup comparisons. Categorical data were described as “number (percentage),” and intergroup comparisons were conducted using the χ^2^ test (chi-square test); for ordinal data (e.g., H–B grading), intragroup changes were analyzed using the Wilcoxon signed-rank test, and intergroup differences were analyzed using the Mann–Whitney *U* test. All statistical tests were two-tailed, with a significance level of α = 0.05 (i.e., *P* < .05 indicated a statistically significant difference). No a priori sample size calculation was performed in this study; however, post hoc analysis based on the primary outcome measure data (reduction in Young modulus of the levator labii superioris: 5.6 ± 1.8 kPa vs 1.0 ± 0.9 kPa) showed a Cohen *d* effect size of 3.41. A total of 31 participants were included in the ultrasound-guided group and 33 in the control group. At a significance level of α = 0.05 (two-tailed independent samples *t*-test), the statistical power of this study reached > 99.9%. This result exceeds the standard power threshold of 80%, indicating that the sample size was sufficient to detect the observed intergroup differences.

### 2.10. High-frequency ultrasound SWE technical principle

High-frequency ultrasound SWE technology may provide a method for precise localization.^[24,25]^ SWE noninvasively, quantitatively, and reproducibly measures tissue Young modulus values, directly reflecting biomechanical properties (e.g., tissue stiffness) and accurately identifying regions of abnormal tissue hardness.^[8,26–28]^

Additionally, ultrasound imaging clearly visualizes superficial facial muscles, nerve pathways, and vascular structures, providing real-time “visual navigation” for acupuncture. Guided by ultrasound, practitioners dynamically monitor needle tip position and insertion depth during acupuncture, ensuring precise arrival at pathological sites while avoiding critical adjacent structures (e.g., facial arteries and parotid ducts), thereby enhancing efficacy and safety.

## 3. Results

### 3.1. Subject screening

**Figure 1.**
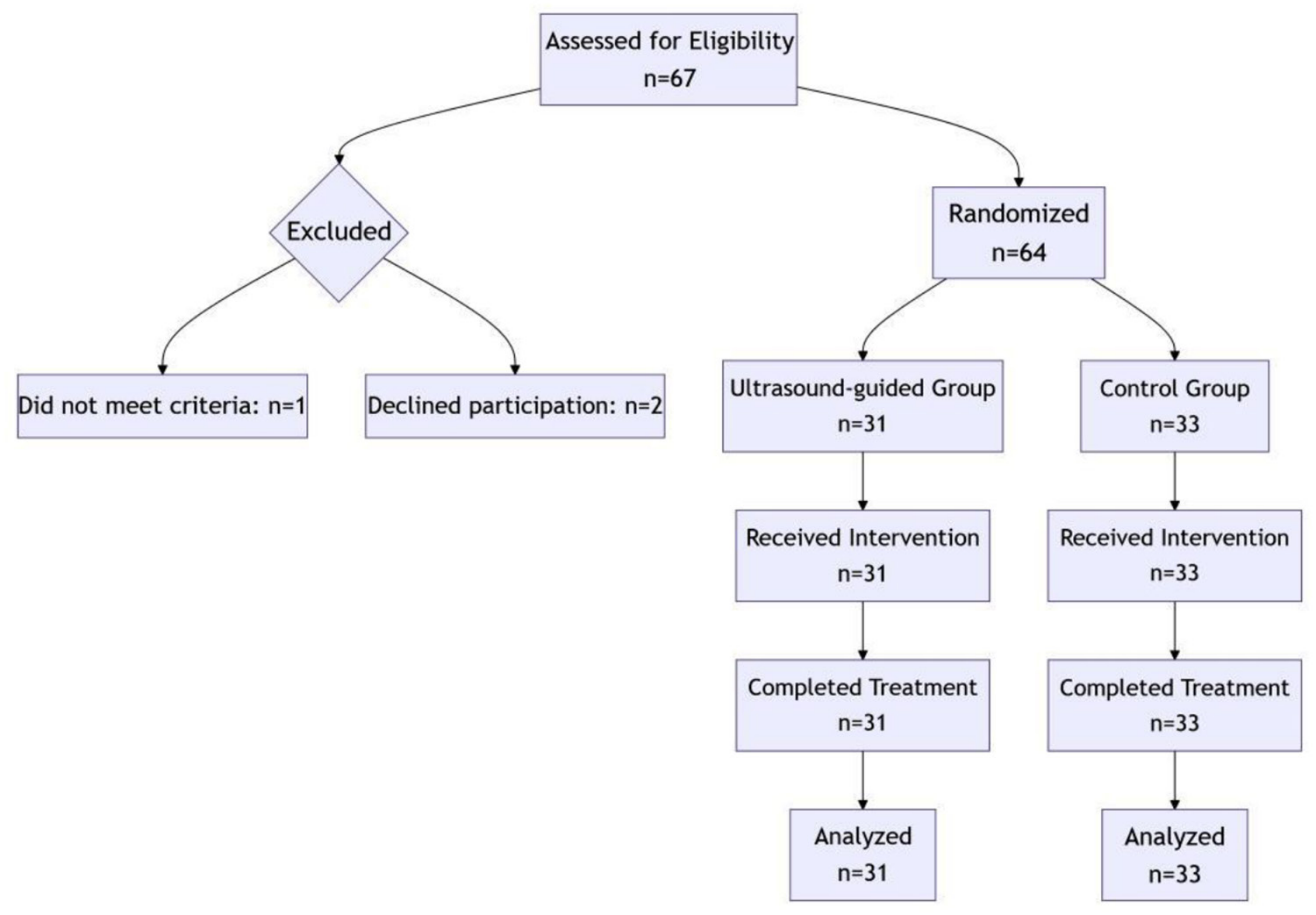
CONSORT flow diagram of participant enrollment, intervention, and analysis. Participant flow: 67 patients with contractural facial synkinesis were screened for eligibility. Three were excluded (1 did not meet inclusion criteria and 2 declined participation), leaving 64 participants who were randomly allocated to either ultrasound-guided group (n = 31) or control group (n = 33). All participants received the allocated intervention and completed the full treatment protocol. Data from all randomized participants were analyzed following the intention-to-treat principle.

A total of 67 patients with facial synkinesis were screened for eligibility. Three patients were excluded (1 patient failed to meet the inclusion criteria due to facial paralysis secondary to postoperative intracranial tumor and 2 patients refused to participate, aged 22 and 58 years, respectively, both with unilateral involvement and disease duration > 3 months), and the remaining 64 participants were randomly assigned to the ultrasound-guided group (n = 31) or the control group (n = 33). All participants received the assigned intervention and completed the entire treatment regimen. This study followed the intention-to-treat principle, and data from all randomized participants were included in the statistical analysis (Fig. 1).

### 3.2. Baseline characteristics of participants

Among the 64 enrolled participants, 31 were assigned to the ultrasound-guided group and 33 to the control group. All baseline evaluation measures, including demographic characteristics, disease-related features, Young modulus of target muscles, H–B grading, and FDI scores, were comparable between the 2 groups (*P* > .05, Table S1, Supplemental Digital Content 1), indicating good comparability. Specifically, there were 30 males (46.9%) and 34 females (53.1%); the age range was 18 to 72 years, with a mean ± SD of 45.2 ± 12.3 years; 35 participants (54.7%) had left-sided involvement, and 29 (45.3%) had right-sided involvement; the disease duration ranged from 3 to 24 months, with a median (interquartile range) of 6.0 (4.0, 12.0) months.

### 3.3. High-frequency ultrasound SWE

**Figure 2.**
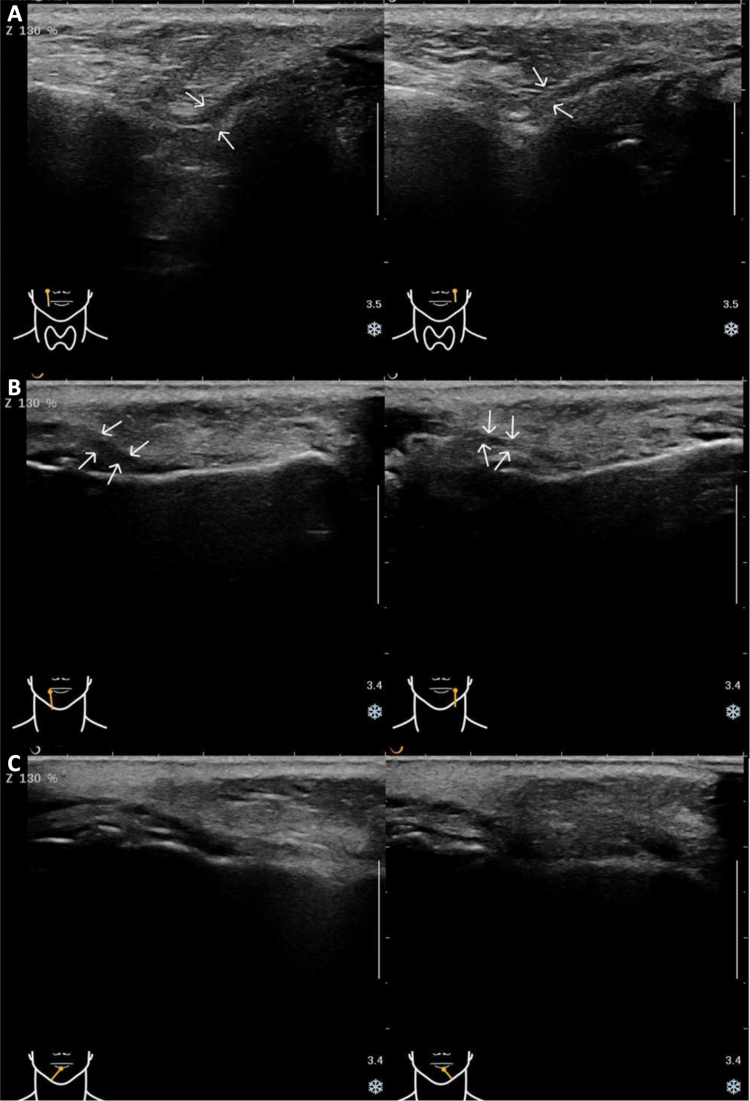
Comparative ultrasound imaging of facial muscles in synkinesis patients. (A) Levator labii superioris: left panel displays healthy side with clear perimysium and right panel shows affected side exhibiting blurred, hyperechoic perimysium. (B) Depressor anguli oris: left panel depicts affected side with blurred, hyperechoic perimysium and right panel presents healthy side with clear perimysium. (C) Depressor labii inferioris: left panel illustrates affected side with blurred, hyperechoic perimysium and right panel demonstrates healthy side with clear perimysium.

We conducted SWE examinations on 30 healthy subjects and 30 patients with facial synkinesis following facial paralysis (Fig. 2). The results showed that Young modulus values of major facial muscle groups in healthy individuals ranged between 6 and 9 kPa. In patients with synkinesis, the unaffected side exhibited clear perimysium and Young modulus values below 10 kPa. Conversely, the affected side showed significantly elevated Young modulus values (mean > 10 kPa), markedly exceeding the normal reference range (6–9 kPa), accompanied by enhanced perimysial echogenicity and structural blurring in the levator labii superioris (Fig. 2A), depressor anguli oris (Fig. 2B), and depressor labii inferioris (Fig. 2C).

### 3.4. Biomechanical improvements quantified by SWE

Before intervention, the baseline Young modulus values of all target muscles were comparable between the 2 groups (*P* > .05, Table S2, Supplemental Digital Content 2, Fig. 4A–C). Table S2, Supplemental Digital Content 2 presents the absolute Young modulus values of target muscles at baseline and post-intervention, while Table S3, Supplemental Digital Content 3 shows the magnitude of reduction in Young modulus (Δ = pretreatment − posttreatment). The 2 tables differ in that Table S2, Supplemental Digital Content 2 reflects static muscle stiffness before and after treatment, whereas Table S3, Supplemental Digital Content 3 quantifies the dynamic therapeutic change in muscle tension. This distinction allows comprehensive evaluation of both baseline status and treatment response.

**Figure 3.**
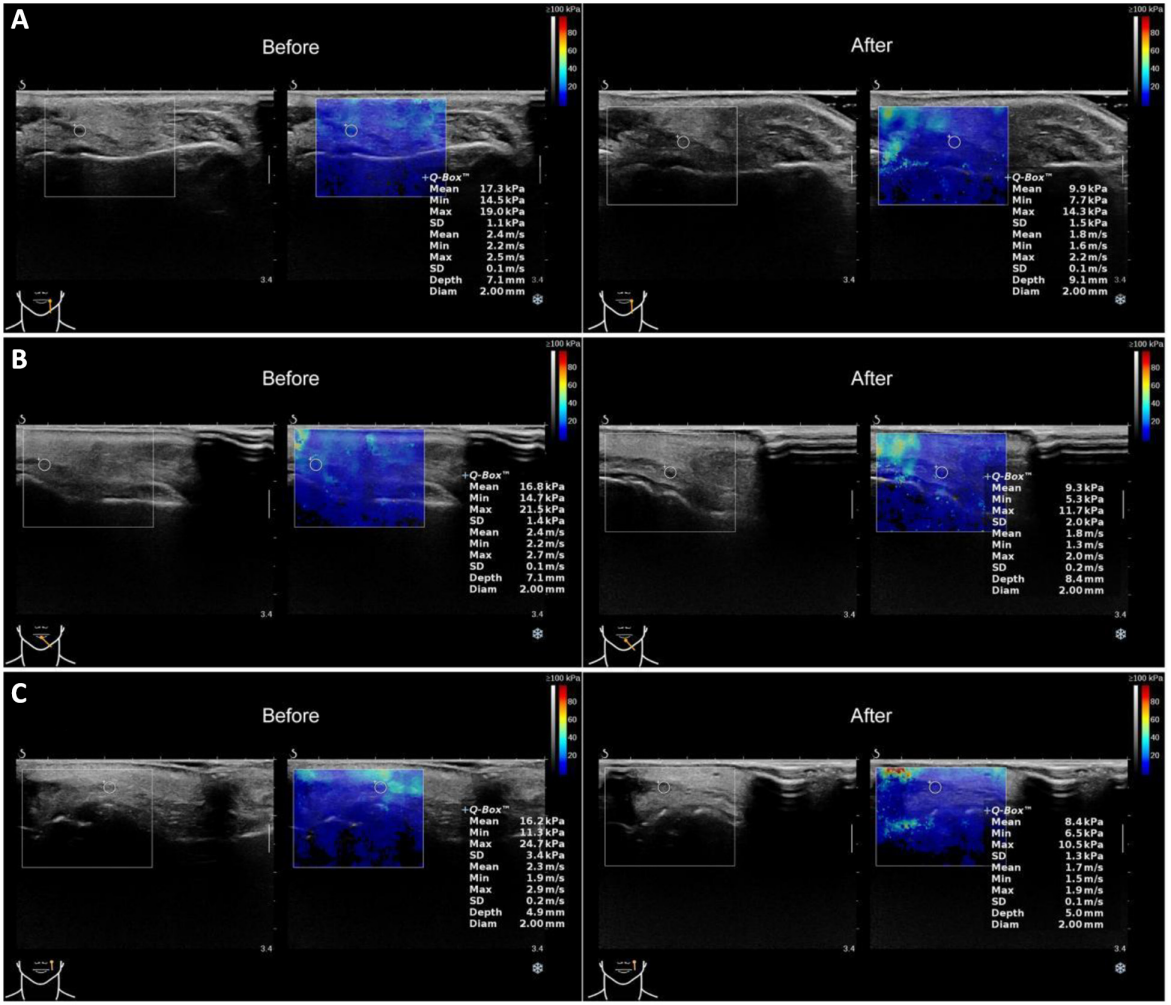
Representative ultrasound and elastographic changes in affected facial muscles of the ultrasound-guided group. (A) Depressor anguli oris: left panel (Pretreatment) demonstrates hyperechoic perimysium with blurred architecture and Young modulus of 18.2 kPa and right panel (Posttreatment) shows normalized perimysial echogenicity with modulus reduced to 9.2 kPa. (B) Depressor labii inferioris: left panel (Pretreatment) exhibits Young modulus of 14.7 kPa and right panel (Posttreatment) reveals reduction to 10.2 kPa. (C) Levator labii superioris: left panel (Pretreatment) displays modulus of 18.6 kPa and right panel (Posttreatment) demonstrates decreased modulus of 13.0 kPa. Dynamic treatment procedure available in Video S1, Supplemental Digital Content 4.

**Figure 4.**
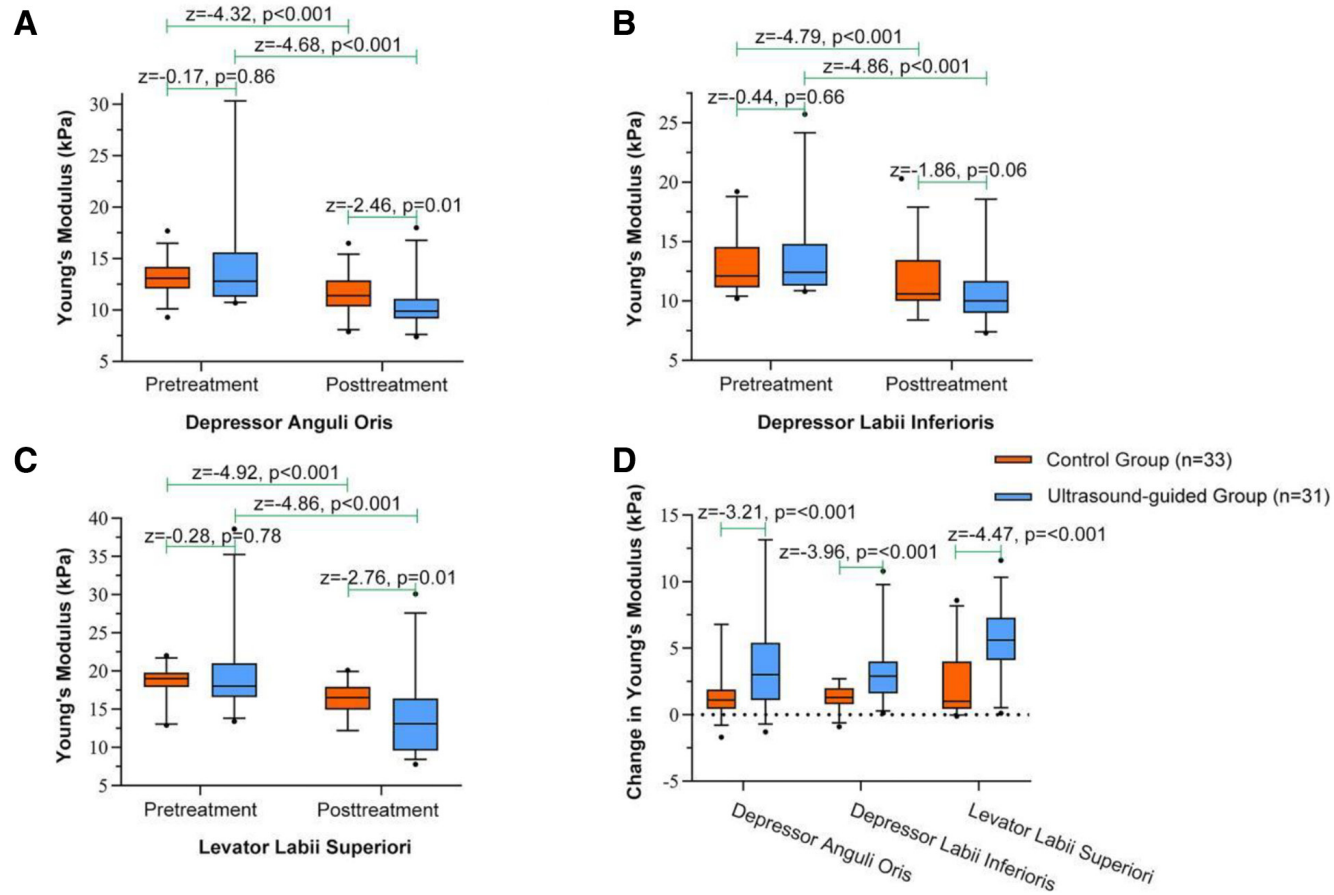
Comparative analysis of Young modulus in facial expression muscles. (A) Depressor anguli oris modulus values pre and posttreatment between groups; (B) Depressor labii inferioris modulus values pre and posttreatment between groups; (C) Levator labii superioris modulus values pre and posttreatment between groups; and (D) ΔYoung modulus (pretreatment–posttreatment difference) for all 3 muscles between groups. Error bars indicate standard deviation throughout.

Intergroup comparisons post-intervention revealed that ultrasound guidance yielded more significant biomechanical improvements: compared with the control group, the ultrasound-guided group exhibited significantly greater reductions in Young modulus of the depressor anguli oris and levator labii superioris muscles (median difference: 3.00 kPa vs 1.10 kPa, *P* = .001; 5.60 kPa vs 1.00 kPa, *P* < .001; Figs. 3A, B, 4A, B, Table S3, Supplemental Digital Content 3). Although the depressor labii inferioris showed a clinically meaningful decreasing trend (median difference: 2.90 kPa vs 1.30 kPa; Figs. 3C, 4C, Table S3, Supplemental Digital Content 3), this difference did not reach statistical significance (*P* = .06).

Intragroup comparisons post-intervention demonstrated that both treatment strategies significantly reduced the muscle stiffness of the depressor anguli oris, depressor labii inferioris, and levator labii superioris muscles (*P* < .001; Table S2, Supplemental Digital Content 2, Fig. 4D). Representative changes in ultrasound and elastography images are shown in Figure 3. After treatment in the ultrasound-guided group, the echo of the perimysium of the target muscles returned to normal, and the Young modulus values decreased significantly.

### 3.5. Functional recovery and clinical efficacy

The results of H–B grading and clinical efficacy before and after treatment in both groups are as following. The pretreatment H–B grade distribution showed that there were no patients with Grade II in either group. In the control group, 57.58% of patients were classified as Grade III, 33.33% as Grade IV, and 9.68% as Grade V. In contrast, the ultrasound-guided group presented a more severe baseline condition, with 38.71% Grade III, 48.39% Grade IV, and 12.90% Grade V at enrollment. After intervention, both groups demonstrated notable improvement in facial nerve function grading. The proportion of patients with mild impairment (Grade II) increased, while the proportion of severe grades (Grade IV and V) decreased in both cohorts. Notably, the ultrasound-guided group exhibited superior therapeutic outcomes. The proportion of Grade II patients in the ultrasound-guided group reached 38.71%, which was significantly higher than that in the control group (21.21%). Meanwhile, the proportion of severe Grade IV cases in the ultrasound-guided group decreased sharply to 6.45%, and no patients remained at Grade V after treatment. In comparison, the control group still retained 9.09% of patients with severe Grade V dysfunction (Table S4, Supplemental Digital Content 5, Fig. 5).

**Figure 5.**
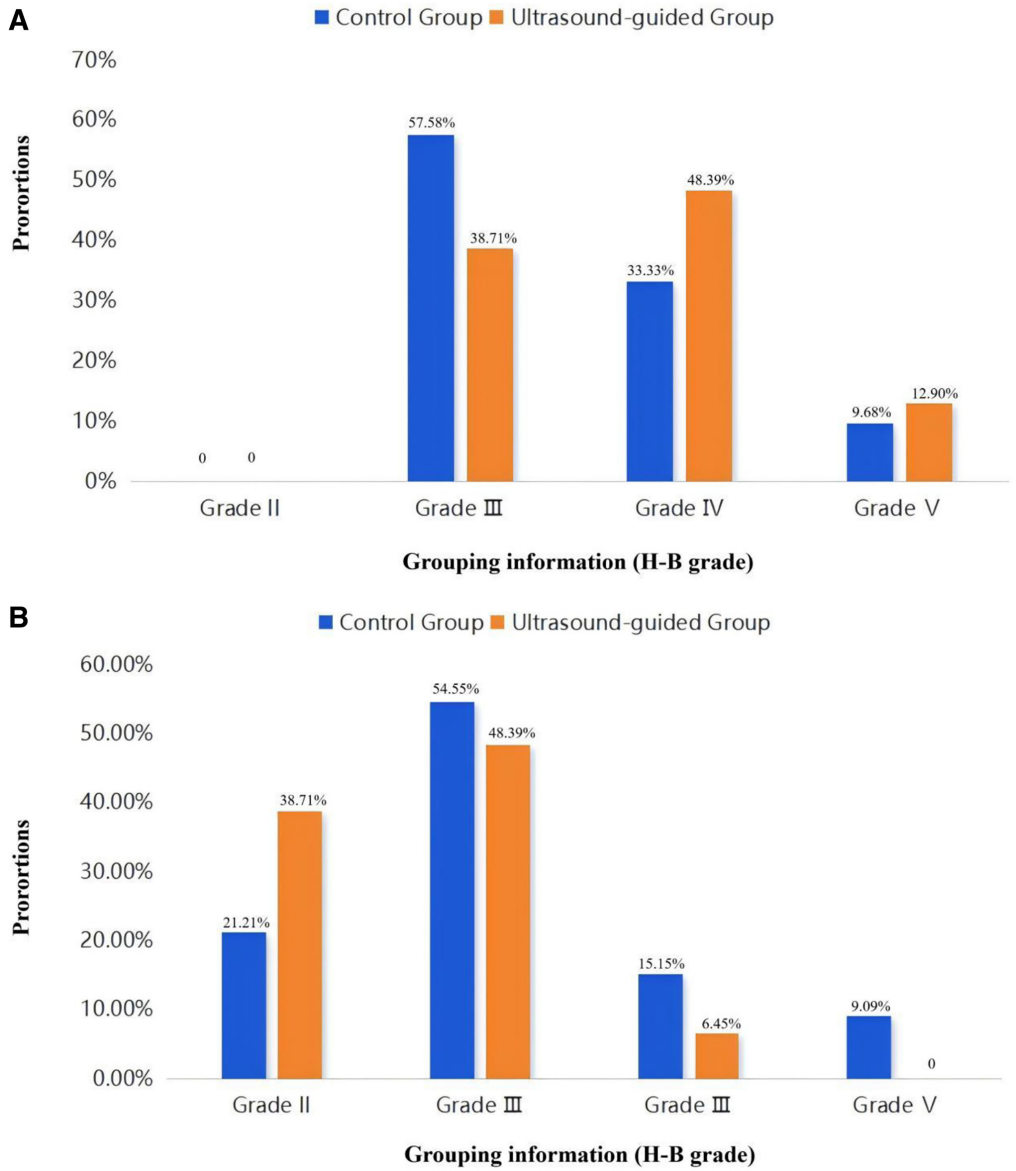
Comparative distribution of House–Brackmann grades pretreatment. (A) Prior to treatment, there was no statistically significant difference in the distribution of H–B grading between the ultrasound-guided group (n = 31) and the control group (n = 33). (B) After treatment, facial nerve function improved in both groups, with a significantly greater improvement in the ultrasound-guided group.

Regarding the total clinical effective rate, the ultrasound-guided group achieved 87.10% (27/31), including 8 cases of marked effectiveness, 19 cases of effectiveness, and 4 cases of ineffectiveness; the control group achieved 42.42% (14/33), including 14 cases of effectiveness and 19 cases of ineffectiveness, with no cured cases in either group (Table 1). The total clinical effective rate of the ultrasound-guided group was significantly higher than that of the control group (χ^2^ = 13.86, *P* < .001).

**Table 1:** Clinical efficacy rates based on House–Brackmann grade improvement among patients with contractural facial synkinesis receiving ultrasound-guided Canggui Tanxue needling technique or conventional acupuncture at the Acupuncture Department of Chongqing Traditional Chinese Medicine Hospital, June 2022 to May 2023.

Group	Control group	Ultrasound-guided group
n	33	31
Cured, n	0	0
Markedly effective, n	0	8
Effective, n	14	19
Ineffective, n	19	4
Total effective rate, %	42.42	87.10

### 3.6. Quality-of-life outcomes

Physical function (FDIP subscale) improved significantly in both groups (both *P* < .001), yet ultrasound guidance provided superior restoration of daily activities. Final FDIP scores were markedly higher in the ultrasound-guided group (74.84 ± 8.42 vs 68.94 ± 13.68, *t* = −2.09, *P* = .04; Table 2), reflecting enhanced oral competence during eating and drinking. For psychosocial impact (FDIS subscale), both groups showed reduced scores (both *P* < .001), but intergroup differences were nonsignificant (*Z* = −0.10, *P* = .92; Table 3).

**Table 2:** Facial Disability Index-physical function (FDIP) scores before and after ultrasound-guided Canggui Tanxue needling technique treatment and conventional acupuncture among patients with contractural facial synkinesis at the Acupuncture Department of Chongqing Traditional Chinese Medicine Hospital, June 2022 to May 2023.

Index	Time point	Control group (Mean ± SD)	Ultrasound-guided group (Mean ± SD)	Statistic (Intergroup)	*P*-value (Intergroup)	Statistic (Intragroup)	*P*-value (Intragroup)
FDIP Score	Pretreatment	65.61 ± 13.57	61.13 ± 7.72	*t* = 1.64	.11	*t* = −4.30	<.001
FDIP Score	Posttreatment	68.94 ± 13.68	74.84 ± 8.42	*t* = −2.09	.04	*t* = −10.99	<.001
n	–	33	31	–	–	–	–

**Table 3:** Facial Disability Index-social function (FDIS) scores before and after ultrasound-guided Canggui Tanxue needling technique treatment and conventional acupuncture among patients with contractural facial synkinesis at the Acupuncture Department of Chongqing Traditional Chinese Medicine Hospital, June 2022 to May 2023.

Index	Time point	Control group (Median [IQR])	Ultrasound-guided group (Median [IQR])p)	Statistic (Intergroup)	*P*-value (Intergroup)	Statistic (Intragroup)	*P*-value (Intragroup)
FDIS Score	Pretreatment	34 (28– 40)	32 (28–52)	*Z* = −0.49	.63	*Z* = −3.65	<.001
FDIS Score	Posttreatment	32 (27– 37)	32 (24–44)	*Z* = −0.10	.92	*Z* = −3.72	<.001
n	–	33	31	–	–	–	–

### 3.7. Safety profile

During the treatment period, 2 cases of mild subcutaneous hematoma occurred in the ultrasound-guided group, with an incidence rate of 6.5%. All hematomas resolved spontaneously within 48 hours after compression treatment. No adverse reactions were observed in the control group. No serious complications such as nerve injury or vascular injury were noted in either group.

## 4. Discussion

### 4.1. Restatement of research objectives and core findings

The present study aimed to precisely locate lesioned muscles using ultrasound SWE combined with the Canggui Tanxue needling technique, explore a safer and more effective targeted treatment strategy, and address the current issues of insufficient precision and lack of objective quantitative evidence in the clinical treatment of facial synkinesis. The core findings of this study are as follows: The ultrasound-guided group exhibited significantly greater reductions in Young modulus of the target muscles (depressor anguli oris and levator labii superioris) compared with the control group; The ultrasound-guided group achieved superior recovery of facial nerve function, with a significantly higher total clinical effective rate than the control group; Patients in the ultrasound-guided group showed better improvements in physical function (FDIP scores) than those in the control group, while there were no statistically significant differences in improvements in psychosocial function (FDIS scores) between the 2 groups. Only 2 cases of mild subcutaneous hematoma occurred in the ultrasound-guided group, indicating good safety.^[29]^ This is consistent with the findings of Bumler et al, who reported that acupuncture-related adverse events (such as subcutaneous hematoma) are mild and transient, with an overall incidence of < 10% in facial acupuncture.^[29]^

### 4.2. In-depth analysis of core findings and comparison with similar studies

#### 4.2.1. Precise improvements in biomechanical indicators of target muscles

In the present study, the median reductions in Young modulus of the depressor anguli oris and levator labii superioris muscles in the ultrasound-guided group were 3.00 and 5.60 kPa, respectively, which were significantly higher than those in the control group (1.10 and 1.00 kPa, *P* ≤ .001). This result indicates that ultrasound-guided Canggui Tanxue needling can more effectively reduce the pathological tension of lesioned muscles.

Among similar studies, Vejbrink et al confirmed that ultrasound technology can improve treatment targeting through ultrasound-guided injection for sequelae of facial paralysis; however, this study focused on injection therapy and did not involve acupuncture intervention.^[30]^ Sauer et al used ultrasound elastography to evaluate facial muscle function in children with facial paralysis and found that the elasticity values of lesioned muscles were significantly increased, but did not explore the effect of ultrasound-guided treatment.^[31]^ The commonality between this study and the aforementioned studies lies in the recognition of the value of ultrasound elastography for quantitative assessment of muscle lesions. The difference is that this study combined real-time SWE navigation with the traditional Canggui Tanxue needling technique, achieving the integration of “assessment-treatment-precise regulation.” The innovation lies in the first clear confirmation of the precise regulatory effect of ultrasound-guided acupuncture on the Young modulus of lesioned muscles, providing quantitative evidence for the biomechanical intervention of facial synkinesis.

Notably, the frontalis and levator anguli oris muscles displayed no such abnormalities on either side. These findings not only indicate that abnormally elevated tension in the levator labii superioris, depressor anguli oris, and depressor labii inferioris is a key pathological feature of synkinesis but also precisely delineate high-tension target sites requiring intervention.

#### 4.2.2. Significant improvements in facial nerve function and clinical efficacy

The ultrasound-guided group showed a more significant improvement in the distribution of facial nerve function assessed by the H–B grading system (*P* = .013), with a total clinical effective rate of 87.1%, which was substantially higher than that of the control group (42.4%, *P* < .001). This therapeutic advantage is prominent among similar acupuncture studies.

treated facial synkinesis with acupuncture regulating the Conception Vessel (Ren Meridian) and Governor Vessel (Du Meridian), achieving a total effective rate of 63.8%.^[32]^ achieved favorable therapeutic outcomes in the treatment of sequelae of facial paralysis by adopting the regimen of contralateral acupuncture combined with thermal moxibustion.^[33]^ With these studies, the significantly higher effective rate in the present study is mainly attributed to the fact that traditional acupuncture relies on meridian syndrome differentiation for acupoint selection, making it difficult to precisely locate hypertonic lesioned muscles. In contrast, this study achieved targeted intervention by real-time locating abnormal areas with Young modulus > 10 kPa via SWE, combined with multidirectional and layered stimulation of the Canggui Tanxue needling technique. The innovation lies in establishing a therapeutic model of “ultrasound-based quantitative localization + precise needling stimulation,” which breaks through the dependence of traditional acupuncture on subjective syndrome differentiation and significantly improves clinical efficacy.

#### 4.2.3. Differential manifestations of patients’ functional recovery

The ultrasound-guided group showed significantly greater improvement in physical function as assessed by FDIP scores (74.84 ± 8.42 vs 68.94 ± 13.68, *P* = .04), whereas there was no statistically significant difference in the improvement of psychosocial function as measured by FDIS scores between the 2 groups (*P* = .92). This result is consistent with the findings of Nellis et al, who pointed out that psychological recovery in patients with facial paralysis often lags behind functional improvement.^[34]^

Compared with the laser acupuncture study by Ton et al, the present study demonstrated superior improvement in physical function but similarly failed to achieve significant enhancement of psychosocial function in the short term.^[35]^ The potential reason for this difference may be that the intervention period of this study was 3 weeks, while the recovery of psychosocial function requires a longer period of functional adaptation and social feedback. The innovation lies in the first quantitative confirmation of the differential effects of ultrasound-guided acupuncture on patients’ physical and psychosocial functions, providing directions for the optimization of subsequent treatment plans. It suggests that psychological intervention should be integrated to promote the comprehensive recovery of patients. This discordance suggests that psychological recovery, particularly social anxiety and self-perception, may lag behind functional gains, requiring extended exposure to improved facial control or adjunctive behavioral interventions.

To facilitate psychological recovery, we recommend a follow-up period of 6 months after the completion of acupuncture treatment, with assessments at 1-, 3-, and 6-month posttreatment using the FDIS scale and generalized anxiety disorder (GAD-7) scale to dynamically monitor changes in psychosocial function. For patients with persistent psychological distress, targeted psychological interventions can be provided, including: cognitive behavioral therapy: to help patients correct negative cognitive biases related to facial appearance and reduce social avoidance behavior; social skills training: to improve communication confidence and interpersonal adaptation through simulated social scenarios; supportive psychotherapy: to provide emotional support and disease-related psychoeducation, alleviating anxiety and depression caused by functional impairment; and group counseling: to create a mutual support platform for patients to share experiences and enhance coping confidence. These interventions can be implemented through a combination of online (teleconsultation) and offline (face-to-face counseling) modes to improve accessibility and compliance.

#### 4.2.4. Manifestation of advantages in treatment safety

Only 2 cases of mild subcutaneous hematoma occurred in the ultrasound-guided group, with an incidence rate of 6.5%. These hematomas healed spontaneously, and no severe neurovascular complications were observed, indicating that the safety profile was comparable to that of the control group.

In traditional facial acupuncture, due to the complex anatomical structure of the face and the abundance of blood vessels and nerves, blind needling may lead to adverse reactions such as hematomas and nerve irritation, with an incidence rate of approximately 10 to 15%.^[36]^ In the present study, real-time ultrasound visualization guidance was used to avoid critical structures such as blood vessels and nerves, significantly reducing the risk of adverse reactions. Compared with Botox injection therapy mentioned in the review by Helmstaedter et al, the present study exhibited superior safety.^[37]^ The innovation lies in integrating ultrasound visualization technology into acupuncture practice, establishing a safety protection mechanism for facial acupuncture, and providing a safety guarantee for acupuncture treatment in high-risk areas.

### 4.3. Study strengths

This study adopts a rigorous single-blind RCT design with adequate allocation concealment, where both outcome assessors and statisticians are blinded to effectively reduce selection and measurement biases while adhering to international RCT standards; it innovates in quantitative indicators by employing SWE-derived Young modulus as the primary outcome measure, enabling objective quantitative evaluation of muscle pathological changes we further compared the findings with those of healthy individuals, establishing a normal reference range of Young modulus (6–9 kPa) for facial muscles and clarifying the pathological characteristics of hypertonic muscles in FSS patients and addressing the limitation of traditional studies relying on subjective scoring systems; it also establishes a novel therapeutic model through the integration of ultrasound navigation with the Canggui Tanxue needling technique, providing a new clinical approach for facial synkinesis management; additionally, real-time ultrasound guidance minimizes the incidence of adverse reactions, significantly enhancing the safety and operability of acupuncture therapy.

### 4.4. Limitations

The present study has some limitations: the follow-up period was short, with only immediate posttreatment efficacy evaluated and no long-term follow-up conducted, thus failing to confirm the durability of biomechanical improvements and the disease recurrence rate; mechanistic research was insufficient, as neurophysiological tests (e.g., electromyography) and neuroimaging examinations were not incorporated, making it impossible to clarify the underlying mechanisms of ultrasound-guided acupuncture in promoting nerve regeneration and cortical remodeling; the sample size was relatively small (n = 64) despite post hoc analysis confirming adequate statistical power, and the single-center design may introduce selection bias, requiring validation of the results’ generalizability through multicenter studies; furthermore, the lack of a placebo control group means the placebo effect of acupuncture itself cannot be completely excluded, which has a certain impact on the objective verification of therapeutic efficacy. Several methodological constraints require Acknowledgments. Firstly, the exclusive assessment of outcomes immediately following the 3-week intervention precludes evaluation of long-term efficacy, leaving the durability of biomechanical improvements and potential recurrence rates undefined. Secondly, while SWE effectively quantified reductions in muscular hypertonicity, the study design did not incorporate neurophysiological investigations to elucidate underlying mechanisms – particularly the relative contributions of peripheral nerve regeneration versus central sensorimotor adaptation to functional recovery. Thirdly, the absence of objective neural metrics, such as electromyographic assessment of axonal integrity or functional MRI mapping of cortical reorganization, limits causal interpretation of the findings between biomechanical normalization and neuroplastic changes. Future investigations should integrate serial assessments extending ≥ 6 months post-intervention and implement multimodal neuroimaging protocols, including diffusion tensor imaging for facial nerve tract reconstruction and task-activated fMRI for quantifying sensorimotor cortex reorganization patterns.

### 4.5. Participant selection and population representativeness

The representativeness of the study population ensures the external validity of RCT results. This study adopted multichannel recruitment (outpatient announcements, medical record screening, community education, and patient follow-up) to avoid selection bias, covering diverse age groups, disease durations, and severity levels consistent with clinical distributions of Chinese FSS patients.^[3,4]^ The included 64 patients (18–72 years, mean 45.2 ± 12.3 years; males 46.9%, females 53.1%) had balanced gender ratio, disease duration of 3 to 24 months (median 6.0 months), and no lateral bias in affected sides (left 54.7%, right 45.3%), which highly matched the target population characteristics.^[3,5]^ Scientific inclusion/exclusion criteria ensured homogeneity while retaining representativeness, and the 0% dropout rate confirmed sample stability. Overall, the enrolled population well represents FSS patients, laying a solid foundation for clinical promotion of the study results.

### 4.6. Influence of participant characteristics on study findings

Key participant characteristics potentially affecting outcomes (age, gender, disease duration, and H–B grading) were balanced between groups (all *P* > .05). Age and disease duration may correlate with nerve/muscle recovery, while gender could impact treatment compliance, but group homogeneity minimized these confounders. Uncollected factors (e.g., smoking history and comorbidity control) might subtly influence results. Future studies could incorporate these variables for stratified analysis to optimize personalized therapy.

## 5. Conclusion

This present study confirms that ultrasound-guided Canggui Tanxue needling is a highly effective, safe, and targeted therapy for FSS. By precisely localizing hypertonic lesioned muscles with a Young modulus > 10 kPa via real-time SWE, this therapy breaks through the limitation of traditional acupuncture relying on subjective syndrome differentiation. It significantly outperforms traditional syndrome differentiation-based acupuncture in reducing pathological muscle tension, improving facial nerve function, and enhancing patients’ physical function, with an overall effective rate of 87.1% and a favorable safety profile – only 6.5% of patients experienced mild subcutaneous hematomas. The present study also found that the recovery of patients’ psychosocial function may lag behind physical function, necessitating long-term follow-up and targeted interventions. In summary, this precise needling technique provides a novel treatment option for patients with abnormal muscle tension in the late stage of facial paralysis, warranting clinical promotion. Future multicenter studies are required to extend the follow-up period, verify its long-term efficacy and recurrence rate, and clarify its mechanism of action by combining neuroelectrophysiological and multimodal imaging technologies.

## Author contributions

**Conceptualization:** Yuan Zhou, Jingxin Mao.

**Formal analysis:** Xi Zhou.

**Funding acquisition:** Yuan Zhou, Jingxin Mao.

**Investigation:** Xiaoming Wu.

**Software:** Linjia Wang.

**Visualization:** Yuan Zhou.

**Writing – original draft:** Yuan Zhou, Jingxin Mao.

**Writing – review & editing:** Jingxin Mao, Yanru Wang.










