# Supplementary material for: A single-blind randomized controlled trial of ultrasound-guided Canggui Tanxue needling technique for contractural facial synkinesis
Source: Medicine (Baltimore). 2026 Jul 17;105(29):e49719. doi: 10.1097/MD.0000000000049719 (PMC13384618; doi:10.1097/MD.0000000000049719)
Supplement: Supplementary file 1 [file medi-105-e49719-s001.docx]

Table S1. Baseline demographic and clinical characteristics of patients with Contractural Facial Synkinesis randomized to Ultrasound-Guided Canggui Tanxue Needling Technique or Conventional Acupuncture at the Acupuncture Department of Chongqing Traditional Chinese Medicine Hospital, June 2022 to May 2023.

| **Group** | **Control group** | **Ultrasound-guided group** | **Test statistic** | ***P*** |
| --- | --- | --- | --- | --- |
| *P* | 33 | 31 | - | - |
| Sex, n (Male/Female) | 14/19 | 13/18 | 0.002 | 0.97 |
| Affected Side, n (Left/Right) | 16/17 | 17/14 | 0.26 | 0.61 |
| Age, years (Mean±SD) | 54.61±11.19 | 49.65±11.19 | 1.77 | 0.08 |
| Disease Duration, months (Mean±SD) | 5.82±1.59 | 5.58±1.91 | 0.54 | 0.59 |
