# Supplementary material for: A single-blind randomized controlled trial of ultrasound-guided Canggui Tanxue needling technique for contractural facial synkinesis
Source: Medicine (Baltimore). 2026 Jul 17;105(29):e49719. doi: 10.1097/MD.0000000000049719 (PMC13384618; doi:10.1097/MD.0000000000049719)
Supplement: Supplementary file 2 [file medi-105-e49719-s002.docx]

Table S2. Young’s modulus values (kPa, median [IQR]) of target facial muscles before and after treatment in patients with Contractural Facial Synkinesis receiving Ultrasound-Guided Canggui Tanxue Needling Technique or Conventional Acupuncture at the Acupuncture Department of Chongqing Traditional Chinese Medicine Hospital, June 2022 to May 2023.

| **Muscle** | **Time Point** | **Control Group**  **(Median [IQR])** | **Ultrasound-guided Group**  **(Median [IQR])** | **Z**  **(Intergroup)** | **P**  **(Intergroup)** | **Z**  **(Intragroup)** | **P**  **(Intragroup)** |
| --- | --- | --- | --- | --- | --- | --- | --- |
| Depressor anguli oris | Pre-treatment | 13.10 (12.10, 14.20) | 12.80 (11.30, 15.60) | -0.17 | 0.86 | -4.32 | <0.001 |
| Depressor anguli oris | Post-treatment | 11.40 (10.35, 12.90) | 9.90 (9.20, 11.10) | -2.46 | 0.01 | -4.68 | <0.001 |
| Depressor labii inferioris | Pre-treatment | 12.10 (11.15, 14.55) | 12.40 (11.30, 14.80) | -0.44 | 0.66 | -4.79 | <0.001 |
| Depressor labii inferioris | Post-treatment | 10.60 (10.00, 13.45) | 10.00 (9.00, 11.70) | -1.86 | 0.06 | -4.86 | <0.001 |
| Levator labii superioris | Pre-treatment | 19.00 (17.90, 19.80) | 18.00 (16.60, 21.00) | -0.28 | 0.78 | -4.92 | <0.001 |
| Levator labii superioris | Post-treatment | 16.50 (14.95, 17.95) | 13.10 (9.60, 16.40) | -2.76 | 0.01 | -4.86 | <0.001 |
| n | — | 33 | 31 | — | — | — | — |
