# Supplementary material for: A single-blind randomized controlled trial of ultrasound-guided Canggui Tanxue needling technique for contractural facial synkinesis
Source: Medicine (Baltimore). 2026 Jul 17;105(29):e49719. doi: 10.1097/MD.0000000000049719 (PMC13384618; doi:10.1097/MD.0000000000049719)
Supplement: Supplementary file 3 [file medi-105-e49719-s003.docx]

Table S3. Reduction magnitude of Young’s modulus (Δ = Pretreatment − Posttreatment, kPa) in target facial muscles after Ultrasound-Guided Canggui Tanxue Needling Technique or Conventional Acupuncture among patients with Contractural Facial Synkinesis at the Acupuncture Department of Chongqing Traditional Chinese Medicine Hospital, June 2022 to May 2023.

| Muscle | Control Group  (Median [IQR]) | Ultrasound-guided Group  (Median [IQR]) | Z | P |
| --- | --- | --- | --- | --- |
| Depressor Anguli Oris | 1.10 (0.45, 1.90) | 3.00 (1.10, 5.40) | -3.21 | <0.001 |
| Depressor Labii Inferioris | 1.30 (0.80, 2.00) | 2.90 (1.60, 4.00) | -3.96 | <0.001 |
| Levator Labii Superioris | 1.00 (0.45, 4.00) | 5.60 (4.10, 7.30) | -4.47 | <0.001 |
