# Supplementary material for: A single-blind randomized controlled trial of ultrasound-guided Canggui Tanxue needling technique for contractural facial synkinesis
Source: Medicine (Baltimore). 2026 Jul 17;105(29):e49719. doi: 10.1097/MD.0000000000049719 (PMC13384618; doi:10.1097/MD.0000000000049719)
Supplement: Supplementary file 5 [file medi-105-e49719-s005.docx]

Table S4. Distribution of House-Brackmann (H-B) grades before and after treatment in patients with Contractural Facial Synkinesis receiving Ultrasound-Guided Canggui Tanxue Needling Technique or Conventional Acupuncture at the Acupuncture Department of Chongqing Traditional Chinese Medicine Hospital, June 2022 to May 2023.

| **Time Point** | **Pretreatment** | | **Posttreatment** | |
| --- | --- | --- | --- | --- |
| Group | Control Group | Ultrasound-guided Group | Control Group | Ultrasound-guided Group |
| *n* | 33 | 31 | 33 | 31 |
| Grade II, n | 0 | 0 | 7 | 14 |
| Grade III, n | 19 | 12 | 18 | 15 |
| Grade IV, n | 11 | 15 | 5 | 2 |
| Grade V, n | 3 | 4 | 3 | 0 |
